# Mechanical or biologic prostheses for mitral valve replacement: A systematic review and meta‐analysis

**DOI:** 10.1002/clc.23854

**Published:** 2022-06-05

**Authors:** Jun Yu, En Qiao, Wei Wang

**Affiliations:** ^1^ Department of Structural Heart Disease, Fu Wai Hospital, National Center for Cardiovascular Diseases, Chinese Academy of Medical Sciences Peking Union Medical College Beijing China

**Keywords:** bioprosthetic, mechanical, mitral valve replacement (MVR)

## Abstract

Either a mechanical or bioprosthetic valve is used in patients undergoing mitral valve replacement (MVR). However, the optimal mitral prosthesis remains controversial. The aim of this meta‐analysis was thus to compare outcomes between mechanical mitral valve replacement (MVRm) and bioprosthetic mitral valve replacement (MVRb) for MVR patients. We searched Embase, PubMed, Web of Science, and Cochrane Library databases from January 1, 2000 to October 31, 2021 for studies that directly compared surgical outcomes of MVRm and MVRb. A total of 22 studies with 35 903 patients were included in the meta‐analysis (*n* = 23 868 MVRm and *n* = 12 035 MVRb). The MVRm group displayed lower long‐term all causes mortality (HR, 0.84; 95% confidence interval [CI]: 0.77−0.91; *p* < .0001; *I*² = 51%), and fewer mitral reoperation (hazard ratio [HR]: 0.34; 95% CI: 0.23−0.50; *p* < .00001; *I*² = 74%) than MVRb group. However, the MVRm group was associated with a greater risk of major bleeding events (HR: 1.21; 95% CI: 1.14−1.29; *p* < .00001; *I*² = 0%), stroke and systemic embolism (HR: 1.20; 95% CI: 1.10−1.32; *p* < .0001; *I*² = 0%) in matched or adjusted data. No significant difference was observed between MVRm and MVRb on operative mortality in matched/adjusted group (risk ratios: 0.83; 95% CI: 0.66−1.05; *p* = .12; *I*² = 0%). The results were consistent with patients aged under 70 years old. Patients who received a MVRm is associated with 16% lower risk of long‐term mortality and 66% lower risk of mitral reoperation, but 20% greater risk of stroke or systemic embolism, 21% greater risk of major bleeding compared with MVRb in matched/adjusted studies group, which were consistent to patients younger than the age of 70 years who underwent MVR.

## 1 INTRODUCTION

Mitral valve disease is the second most common valvular lesion, preceded by aortic stenosis.^1^ Mitral valve replacement (MVR) is still commonly performed for primary and secondary (or functional) mitral valvulopathy, especially in patients with severe, symptomatic mitral valve disease unsuitable for surgical repair.^2^

The mechanical and bioprosthetic valves can be used for MVR. However, the optimal choice of prosthetic valves, in terms of age or prosthesis position, in MVR surgery remains unclear.^3^ The European Society of Cardiology suggested surgery with mechanical valves for patients under 65 years while bioprosthesis for patients older than 70 years; The American Heart Association recommend a mechanical valve prosthesis in patients under 50 years and a biologic valve over 70 years.^4^ Between these ages, recent consensus guidelines recommend the selection of prosthetic valves based on patient preferences and expert advice, and suggested that it should be a shared decision‐making process.^2^ Therefore, age appears to be the most objective factor to be considered in determining the prosthetic types.

Each type of prosthetic valve has associated with benefits and risks. Mechanical valves are favored in younger patients who consequently face a much lower lifetime risk of reoperation, or in patients who had a lifetime risk of reoperation. Still, the increased risk of bleeding and stroke with mandatory lifelong anticoagulation, which limited the quality of life, are significant disadvantages. Bioprosthetic valves are recommended in patients who show poor compliance with anticoagulant treatment, but biologic structural valve deterioration has an accelerated course in patients' mitral reoperation. Therefore, older patients may be more appropriate to select a bioprosthetic valve. However, advances in transcatheter MVR or repair technology have made it an attractive alternative reoperation approach to conventional open‐heart surgery and might improve the use of bioprosthetic valves in younger patients.^5^

Two randomized clinical trials were conducted in the 1970s and 1980s to compare survival and outcomes in patients receiving a mechanical or bioprosthetic valve in the mitral position that showed no significant difference in late survival.^6^, ^7^ However, these trials did not focus on any specific age group. The two most extensive retrospective studies of MVR in patients aged 50−69 years showed conflicting results. Among 3433 patients aged 50−69 years undergoing MVR in New York State, Chikwe et al.^8^ reported no significant survival difference at 15 years in 664 patients matched by propensity score (hazard ratio [HR]: 0.95; 95% confidence interval [CI]: 0.79−1.15; *p* = .62). On the contrary, among 8015 patients aged 50−69 years undergoing MVR in California, Goldstone et al.^9^ reported bioprosthesis was associated with significantly higher mortality than mechanical prosthesis (HR: 1.16; 95% CI: 1.04−1.30; *p* = .01) in inverse probability weighting data. Therefore, the optimal prosthesis selection for MVR patients under 70 remains controversial. Recent studies differ in their results. Kaneko et al.^10^, Hu et al.^11^, and Chen et al.^12^ favored mechanical mitral valve replacement (MVRm) over bioprosthetic mitral valve replacement (MVRb) for long‐term survival. Cetinkaya et al.^13^, Cen et al.^14^, and Bernard et al.^15^ suggested that the type of prosthesis did not influence on long‐term survival. This meta‐analysis was performed to evaluate the clinical outcomes between mechanical and biologic valves in MVR patients. We also analyzed patients under the age of 70 to provide information regarding valve choice in this age group. Subgroups of matched/adjusted and unmatched/unadjusted studies were also analyzed for exploring the source of statistical heterogeneity and a more detailed comparison.

### 1.1 Methods

We conducted the systematic review and meta‐analysis based on the Cochrane Handbook for Systematic Reviews and Interventions^16^ and reported results following the Preferred Reporting Items for Systematic Reviews and Meta‐Analyses and Meta‐Analysis of Observational Studies in Epidemiology guidelines. Institutional Review Board approval was waived due to the nature of the study. The study was registered on PROSPERO (CRD42021279740).

### 1.2 Search strategy

We searched electronic databases of Embase, PubMed, Web of Science, and Cochrane Library systematically to identify relevant research published from January 1, 2000 to October 31, 2021, that compared the clinical outcomes of the mechanical and biologic prosthesis in patients undergoing MVR. The following key text word was used either alone or in combination: “mechanical mitral valve” and “biological mitral valve.” The bibliographies of included studies were manually scrutinized for further identification of potentially relevant studies according to predefined selection criteria.

### 1.3 Eligibility criteria

The inclusion criteria were as follows: (I) direct comparison of mechanical versus biological valves; (II) studies reported at least one comparative clinical outcome, including operative mortality, long‐term mortality, major bleeding, stroke or systemic embolism, mitral valve reoperation or information with sufficient detail to facilitate the extraction of the HR, standard errors (SE), or Kaplan−Meier curves. When institutions reported duplicate studies with sample overlap, only the most updated study included. All studies were limited to the English language. Reviews, conference abstracts, case reports were excluded in this meta‐analysis.

### 1.4 Data extraction and quality assessment

Two authors independently reviewed each article and extracted data from tables, figures, and texts. Any discrepancies between the two investigators were resolved by consensus or a discussion with the professor. The quality of each study was assessed using the Newcastle Ottawa Scale (NOS). A maximum of 9‐points were allocated to each individual study according to three main dimensions: 4 for study group selection, 2 for comparability between groups, and 3 for ascertainment of outcomes. A study with a NOS score of 6 or higher was deemed as high quality.

### 1.5 Statistical analysis

For baseline demographics, we used mean difference (MD) and risk ratio (RR) through an inverse variance method to pool continuous and binary characteristics, respectively. RR for operative mortality, and HR for long‐term mortality, bleeding, stroke or systemic embolism, and mitral reoperation were pooled on the logarithmic scale using the generic inverse variance method from the individual studies. When the HR was not provided, instead of Kaplan–Meier survival curves, we acquired an estimated HR and its variance from such curves through a calculation spreadsheet developed by Tierney et al.^17^ We assessed statistical heterogeneity using a Q‐statistic and *I*² test. The estimated values of *I*² < 25%, 25% < *I*² < 50%, and *I*² > 50% corresponded to low, moderate, and high degrees of heterogeneity, respectively. A random effects model was used when significant heterogeneity is present (*p* < .1 or *I*² > 50%). Subgroups were created for propensity score‐matched or risk‐adjusted data versus unmatched/unadjusted data, and we used only the papers enrolling patients aged less than 70 years for subgroup analysis. Studies that reported both matched/risk‐adjusted and unmatched/unadjusted data were included separately for subgroup comparisons. Sensitivity analyses were performed by omitting each study in sequence. Funnel plots were assessed for publication bias. Meta‐analysis was conducted using Review Manager (RevMan version 5.4; The Cochrane Collaboration, 2020).

### 1.6 Meta‐regression

A univariate meta‐regression on long‐term mortality and mitral reoperation was conducted for potential confounding bias from observational studies. These two outcomes were analyzed because of significant differences, high heterogeneity, and the adequate studies. Eight covariates were introduced for the meta‐regression: mean age, sex of male, coronary artery disease (CAD), hypertension, diabetes mellitus (DM), atrial fibrillation (AF), kidney disease (KD), concomitant coronary artery bypass grafting. Meta‐regression was performed using a mixed‐effects model with “meta” package. The model used the restricted maximum likelihood as a heterogeneity estimator and the Hartung–Knapp–Sidik–Jonkman method to adjust the SE of estimated coefficients as recommended.^18^ Meta‐regression process was performed with R software, version 4.1.0 (R Foundation, available at: www.r-project.org).

## 2 RESULTS

### 2.1 Characteristics of included studies

The study selection process is summarized in Supporting Information: Figure 1. Eventually, there were 22 studies^8^, ^9^, ^10^, ^11^, ^12^, ^13^, ^14^, ^15^, ^19^, ^20^, ^21^, ^22^, ^23^, ^24^, ^25^, ^26^, ^27^, ^28^, ^29^, ^30^, ^31^, ^32^ that met the eligibility criteria for meta‐analysis, one of which was nonrandomized prospective study^26^ and 21 of which were retrospective cohort studies. The studies involved 35 903 patients (*n* = 23 868 MVRm and *n* = 12 035 MVRb) who underwent MVR enrolled from 1969 to 2020. The characteristics of individual studies are summarized in Table 1. Seven studies^9^, ^21^, ^23^, ^24^, ^28^, ^29^, ^32^ reported both mitral and aortic valve replacement cohorts, and we only extracted MVR parts from these studies. For one large study^9^ of these, we included data from patients aged 40−49, 50−69, and 70−79 years, respectively. And a small study^28^ reported on patients with preoperative renal failure (15 MVRm vs. 34 MVRb). One single‐center nonrandomized prospective study^26^ included only young female patients who underwent MVR. To be maximally inclusive, all studies were included in the primary analysis. Sensitivity analyses excluding one of each study in sequence did not change any pooled results. The quality assessment showed a NOS score of 6 or higher for all studies with a mean NOS score of 6.9, indicating the presence of high methodological quality.

**Table 1:** Study characteristics

						Overall cohort						Matched/adjusted cohort						
Study	Country	Study year	Follow‐up in years (mean or median)		Age (year)	Number of patients included		Mean age (year)		Male		Number of patients included		Mean age (year)		Male		NOS score
			MVRm	MVRb		MVRm	MVRb	MVRm	MVRb	MVRm	MVRb	MVRm	MVRb	MVRm	MVRb	MVRm	MVRb	
Chikwe (2015)	America	1997−2007	8.2		50−69	2638	795	59.7 ± 5.7	61.2 ± 5.9	1052	340	664	664	61.0 ± 5.5	60.8 ± 5.9	282	276	8
Schnittman (2018)	America	1997−2006	12.4		18−50	2345	382	43 [38–47]	42 [36–47]	933	143	373	373	42 [36–47]	42 [36–47]	140	134	8
Kaneko (2014)	America	1991−2012	7.0		<65	627	141	51.8 ± 9.7	54.3 ± 10.3	268	63	125	125	53.2 ± 9.0	53.8 ± 10.6	76	82	7
Hu (2020)	China	2002−2018	8.7		50−69	562	136	58.7 ± 5.2	62.2 ± 4.1	339	81	369	123	59.9 ± 4.2	60.6 ± 3.9	237	77	8
Chen (2021)	China	2000−2013	6.2	3.1	<65	2563	1075	54.2 [46.6−62.2]	65.5 [56.8−72.2]	975	372	788	788	61.2 [53.8−67.8]	61.8 [53.3−69.6]	280	275	8
Sultan (2019)	America	2011−2017	2.9		all	306	522	58.2 ± 11.8	70.3 ± 12.3	131	222	225	225	‐	‐	‐	‐	7
Cetinkaya (2019)	Germany	2005−2015	6.4		all	59	265	56.0 [50.0–62.0]	71.0 [62.0−77.0]	35	128	‐	‐	‐	‐	‐	‐	7
Goldstone (2017) (40−49 years)	America	1996−2013	7.6	4.6	40−49	1340	292	45.6 ± 2.8	45.8 ± 3.0	654	130	1341.8	285.9	45.7 ± 2.8	45.5 ± 3.0	646.1	139.0	8
Goldstone (2017) (50−69 years)					50−69	5714	2301	60.7 ± 5.6	62.4 ± 5.5	2787	1180	5748.9	2278.2	61.2 ± 5.6	61.1 ± 5.8	2858.0	1119.1	
Goldstone (2017) (70−79 years)					70−79	2928	2928	74.6 ± 2.8	75.1 ± 2.8	1327	1344	2937.5	2919.9	74.9 ± 2.8	74.9 ± 2.8	1347.3	1336.5	
Ribeiro (2014)	Brazil	1990−2008	8.9		all	247	105	50.8 ± 12.5	55.8 ± 14.9	116	49	‐	‐	‐	‐	‐	‐	7
Thourani (2011)	America	1996−2008	4.5		20−83	15	34	45.9 ± 15.4	53.9 ± 12.1	9	23	‐	‐	‐	‐	‐	‐	6
Toyoda (2018)	America	1998−2010	6.8		all	874	729	54.0 ± 13.7	61.4 ± 14.7	506	394	‐	‐	‐	‐	‐	‐	8
Cen (2001)	America	1976−1995	6.0	12.0	all	644	495	61.0 [50.0−69.0]	62.0 [52.0−69.0]	213	208	‐	‐	‐	‐	‐	‐	6
Mosa (2016)	Saudi Arabia	1999−2012	5.2		all	145	50	46.1 ± 11.5	53.7 ± 16.4	58	13	‐	‐	‐	‐	‐	‐	6
Kulik (2006)	Canada	1977−2002	5.5		50−65	222	49	‐	‐	‐	‐	‐	‐	‐	‐	‐	‐	6
Khan (2001)	America	1976−1999	‐	‐	≥18	513	402	‐	‐	‐	‐	‐	‐	‐	‐	‐	‐	6
Fino (2018)	Canada	2007−2013	3.0		≤70	45	41	63.1 ± 4.4	63.8 ± 3.8	31	29	‐	‐	‐	‐	‐	‐	6
Singab (2020)	Egypt	2010−2020	‐	‐	<70	181	174	46.5 ± 10.0	47.6 ± 9.9	0	0	‐	‐	‐	‐	‐	‐	6
Prasongsukarn (2005)	Canada	1982–1998	5.2	6.5	61−70	312	353	66.2 ± 2.8	66.5 ± 3.0	159	150	‐	‐	‐	‐	‐	‐	7
Yao (2003)	Japan	1973−1998	5.6	8.6	19−76	125	154	55.1 ± 9.7	49.3 ± 11.8	42	48	‐	‐	‐	‐	‐	‐	6
Bernard (2021)	Canada	2000−2016	‐	‐	all	236	188	64.1 ± 9.3	72.0 ± 7.2	160	141	126	126	69.2 ± 7.0	69.2 ± 6.6	86	85	8
Kim (2018)	South Korea	1997−2015	10.9		>17	1134	303	52.4 ± 10.3	60.2 ± 13.2	382	89	‐	‐	‐	‐	‐	‐	7
Ruel (2007)	Canada	1969−2004	13.4		<60	93	121	48.2 ± 9.2	48.9 ± 8.2	40	41	‐	‐	‐	‐	‐	‐	6

Abbreviations: MVRb, bioprosthetic mitral valve replacement; MVRm, mechanical mitral valve replacement; NOS, Newcastle Ottawa Scale.

### 2.2 Baseline characteristics of included patients (Table 2)

**Table 2:** Baseline characteristics of included patients

	Matched/adjusted studies			Unmatched/unadjusted studies		
	RR or MD,(95%CI)	95% CI	*p* Value	RR or MD,(95%CI）	95% CI	*p* Value
Characteristics						
Age, year	0.02	−0.1 to 0.14	.74	−3.07	−3.96 to −2.19	<.00001
Sex, male, %	1.01	0.98−1.04	.64	1.02	0.97−1.06	.77
NYHA class III/IV, %	0.99	0.88−1.11	.83	1.03	0.93−1.14	.9
Comorbidity						
Coronary artery disease, %	1.00	0.98−1.01	.87	0.84	0.76−0.93	.0004
Hypertention, %	1.00	0.97−1.03	.93	0.89	0.82−0.96	.01
Diabetes mellitus, %	0.99	0.93−1.05	.65	0.79	0.70−0.90	.003
Atrial fibrillation, %	1.00	0.97−1.04	.88	0.99	0.88−1.11	.79
Chronic kidney disease/renal failure, %	1.03	0.94−1.13	.53	0.72	0.60−0.85	.0002
Echocardiographic variables						
LVEF, %	−0.20	−3.63 to 3.23	.91	0.88	−0.19 to 1.95	.11
LA, mm	‐	‐	‐	0.11	−0.02 to 0.23	.1
Concomitant procedure						
CABG, %	0.96	0.92−1.00	.03	0.82	0.71−0.95	.03
Tricuspid procedure, %	0.93	0.80−1.07	.32	0.89	0.75−1.05	.34

Abbreviations: CABG, coronary artery bypass grafting; CI, confidence interval; LA, left atrium; LVEF, left ventricular ejection fraction; MD, mean difference of mechanical versus biological valves; NYHA, New York Heart Association; RR, risk ratio of mechanical versus biological valves.

In unmatched/unadjusted studies group, patients with MVRm were significantly younger (MD: −3.07; 95% CI: −3.96 to −2.19 years; *p* < .00001), and significantly lower incidence of CAD (RR: 0.84; 95% CI: 0.76−0.93; *p* = .0004), hypertension (RR: 0.89; 95% CI: 0.82−0.96; *p* = .01), DM (RR: 0.79; 95% CI: 0.70−0.90; *p* = .003), chronic KD or renal failure (RR: 0.72; 95% CI: 0.60−0.85; *p* = .0002), less concomitant CABG (RR: 0.82; 95% CI: 0.77−0.95; *p* = .03) compared with patients with MVRb. In patients with lower LVEF (MD: 0.88; 95% CI: −0.19 to 1.95; *p* = .11) and smaller LA (MD: 0.11; 95% CI: −0.02 to 0.23; *p* = .1), there seems a trend toward less use of mechanical valves. There were no differences of baseline characteristics in the matched/adjusted studies group, but concomitant CABG procedure exhibited nuances between MVRm and MVRb (RR: 0.96; 95% CI: 0.92−1.00; *p* = .03).

### 2.3 Operative mortality

**Figure 1:**
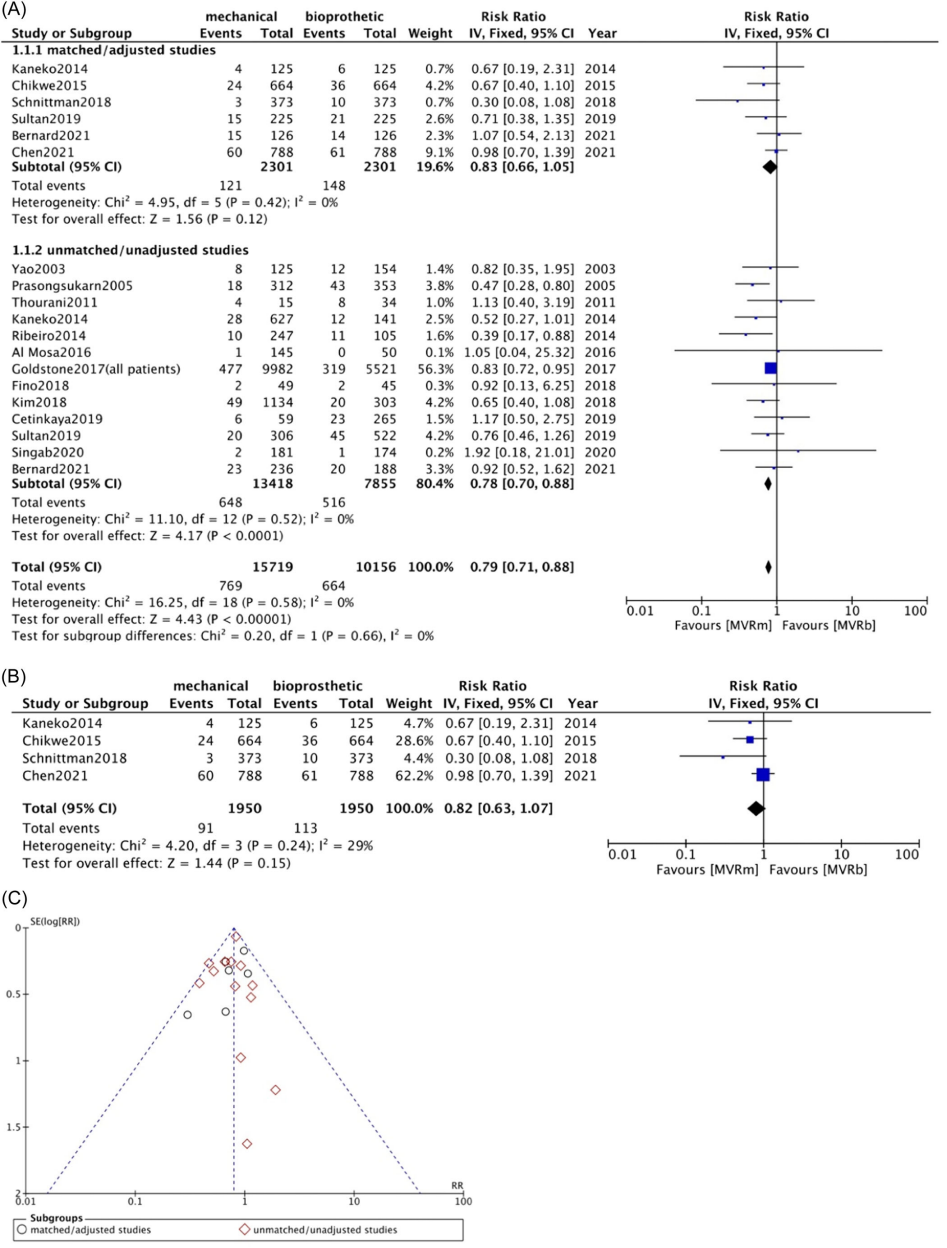
Meta analysis for operative mortality. (A) Forest plot for operative mortality between MVRm and MVRb; (B) Forest plot for operative mortality between MVRm and MVRb for patients aged less than 70 years in matched/adjusted studies; (C) Funnel plot for operative mortality between MVRm and MVRb. CI, confidence interval; MVRm, mechanical mitral valve replacement; MVRb, bioprosthetic mitral valve replacement.

Thirteen studies^9^, ^10^, ^13^, ^15^, ^19^, ^20^, ^22^, ^26^, ^27^, ^28^, ^30^, ^31^, ^32^ in unmatched/unadjusted group and six studies^8^, ^10^, ^12^, ^15^, ^25^, ^27^ in matched/adjusted group provided information on operative mortality, as defined by all causes mortality occurring withing 30 days after surgery or in‐hospital death, which was significant lower in patients receiving MVRm in unmatched/unadjusted group (RR: 0.78; 95% CI: 0.70−0.88; *p* < .0001; *I*² = 0%; Figure 1A). But no significant difference was observed in matched/adjusted group with no statistical heterogeneity (RR: 0.83; 95% CI: 0.66−1.05; *p* = .12; *I*² = 0%; Figure 1A). Four studies^8^, ^10^, ^12^, ^25^ of patients aged less than 70 years in matched/adjusted group showed no significant difference of MVRm and MVRb in operative mortality (RR: 0.82; 95% CI: 0.63−1.07; *p* = .15; *I*² = 29%; Figure 1B).

### 2.4 Long‐term mortality

Fourteen studies^8^, ^10^, ^13^, ^14^, ^15^, ^19^, ^21^, ^23^, ^24^, ^25^, ^29^, ^30^, ^31^, ^32^ in unmatched/unadjusted group and 14 studies^8^, ^9^, ^10^, ^11^, ^12^, ^13^, ^14^, ^15^, ^19^, ^25^, ^27^, ^28^, ^29^, ^30^ in matched/adjusted group documented details on long‐term mortality. The results indicated that patients in MVRm group exhibited a significant lower long‐term risk of death both in unmatched/unadjusted group (HR: 0.77; 95% CI: 0.70−0.84; *p* < .00001; *I*² = 40%; Figure 2A) and matched/adjusted group (HR: 0.84; 95% CI: 0.77−0.91; *p* < .0001; *I*² = 51%; Figure 2A). The meta regression (Table 3) demonstrated that mean age (*β* = .0153, *p* = .0022), CAD (*β* = .007, *p* = .0234), hypertension (*β* = .007, *p* = .0234), DM (*β* = .0284, *p* = .0224) were the main covariates affecting long‐term mortality. It also revealed that male (*β* = −0.096, *p* = .909), AF (*β* = .0067, *p* = .2406), KD (*β* = .0039, *p* = .870) had no significant effect on long‐term mortality between two implants (Supporting Information: Figure 2A). In a subgroup analysis of patients less than 70 years old,^8^, ^9^, ^10^, ^11^, ^12^, ^14^, ^25^ matched/adjusted long‐term mortality (HR: 0.77; 95% CI: 0.68−0.88; *p* < .0001; *I*² = 58%; Figure 2B) was lower with MVRm.

Figure 2Meta analysis for long‐term mortality. (A) Forest plot for long‐term mortality between MVRm and MVRb; (B) Forest plot for long‐term mortality between MVRm and MVRb for patients aged less than 70 years in matched/adjusted studies; (C) Funnel plot for long‐term mortality between MVRm and MVRb. CI, confidence interval; MVRb, bioprosthetic mitral valve replacement; MVRm, mechanical mitral valve replacement.

**Figure:**
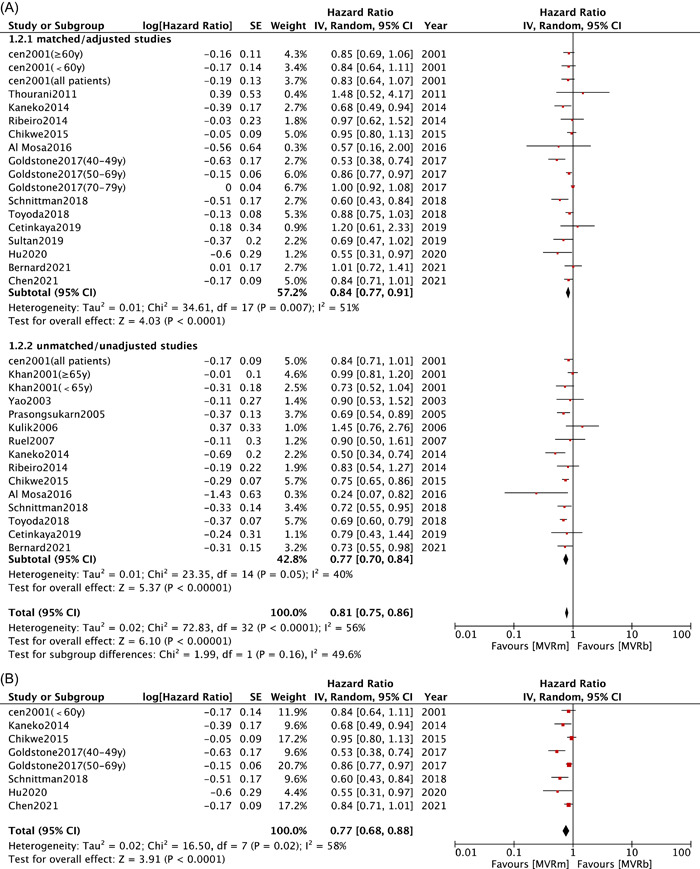

**Figure:**
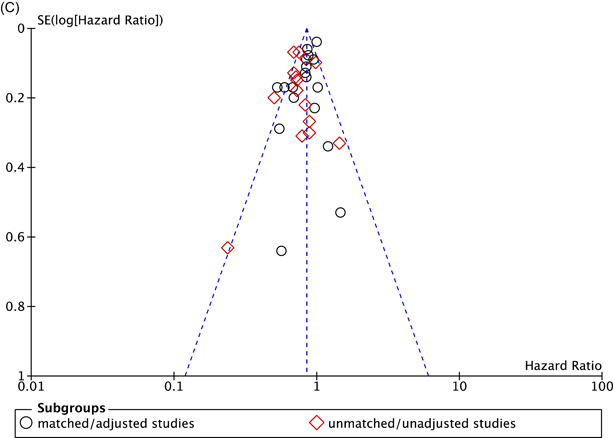

**Table 3:** Meta‐regression output

Model	*N*	Long‐term mortality				*N*	Mitral reoperation			
		Coeff. (SE)	Tau²	*I*² (%)	*p* Value		Coeff. (SE)	Tau²	*I*² (%)	*p* Value
Univariate										
Mean age	9	0.01530 (0.0033)	0.0005	4.17	.0022	7	0.0327 (0.0129)	0.0000	0.01	.0523
Sex, male	9	−0.0960 (0.8104)	0.0424	83.51	.9091	7	0.0113 (0.0357)	0.2883	82.62	.7649
Coranary artery disease	8	0.0070 (0.0023)	0.0003	3.31	.0234	6	0.0260 (0.0107)	0.0000	0.01	.0716
Hypertension	8	0.0117 (0.0037)	0.0034	29.22	.0199	7	0.0346 (0.0094)	0.0000	0.01	.0140
Diabetes mellitus	8	0.0284 (0.0093)	0.0064	46.79	.0224	7	0.0911 (0.0348)	0.0364	37.11	.0473
Atrial fibrillation	8	0.0067 (0.0052)	0.0368	82.13	.2406	7	0.0098 (0.0183)	0.2216	78.55	.6137
Kidney disease	9	0.0039 (0.0228)	0.0441	83.38	.8700	7	0.1047 (0.0854)	0.1550	71.79	.2747
Con‐CABG	7	0.0062 (0.0029)	0.0145	61.03	.0888	5	0.0240 (0.0016)	0.0000	0.00	.0006

Abbreviations: Coeff., Coefficient; Con‐CABG, concomitant coronary artery bypass grafting; SE, standard error.

### 2.5 Stroke or systemic embolism

Six studies^8^, ^9^, ^11^, ^12^, ^15^, ^25^ in matched/adjusted group demonstrated that patients in MVRm significantly increased the risk of stroke or systemic embolism with no heterogeneity among studies (HR: 1.20; 95% CI: 1.10−1.32; *p* < .0001; *I*² = 0%; Figure 3A). However, patients of six studies^8^, ^10^, ^21^, ^23^, ^25^, ^31^ in unmatched/unadjusted group showed no difference on stroke or systemic embolism between MVRm and MVRb, whereas the heterogeneity was high (HR: 1.14; 95% CI: 0.72−1.79; *p* = .58; *I*² = 73%; Figure 3A). When we performed a sensitivity analysis by removing the data reported by Yao et al.^31^, the heterogeneity was deemed more acceptable, and the results were pooled by the rest of the studies indicated a significant difference in stroke or systemic embolism between MVRm and MVRb (HR: 1.37; 95% CI: 1.04−1.79; *p* = .02; *I*²= 25%; Figure 3B). Matched/adjusted stroke or systemic embolism were higher for patients less than 70 years of age in MVRm group (HR: 1.24; 95% CI: 1.10−1.40; *p* = .0005; *I*²= 0%; Figure 3C).^8^, ^9^, ^11^, ^12^, ^25^

Figure 3Meta analysis for stroke or systemic embolism. (A) Forest plot for stroke or systemic embolism between MVRm and MVRb. (B) Forest plot for stroke or systemic embolism between MVRm and MVRb (sensitivity analyses by omitting Yao^31^). (C) Forest plot for stroke or systemic embolism between MVRm and MVRb for patients aged less than 70 years in matched/adjusted studies; (D) Funnel plot for stroke or systemic embolism between MVRm and MVRb. CI, confidence interval; MVRb, bioprosthetic mitral valve replacement; MVRm, mechanical mitral valve replacement.

**Figure:**
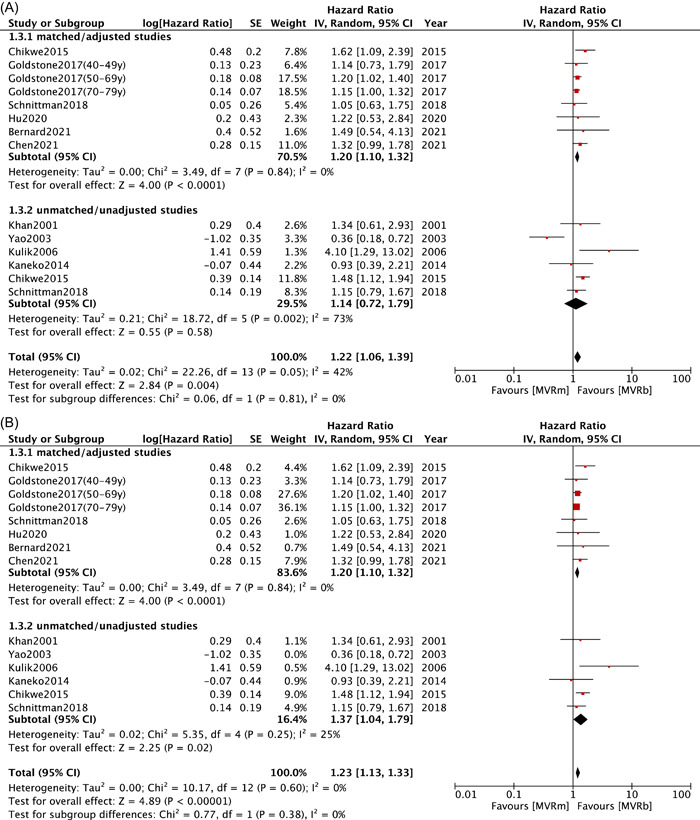

**Figure:**
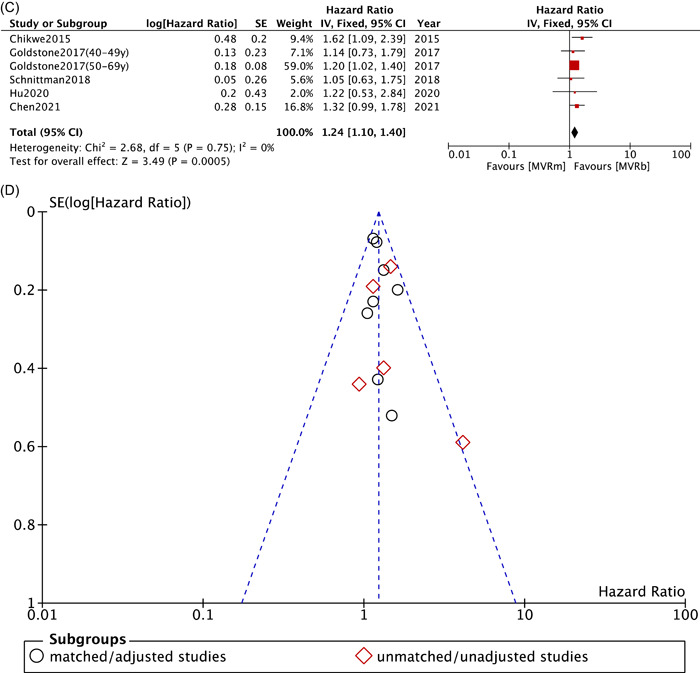

### 2.6 Major bleeding events

Analysis of major bleeding was based on data obtained from six studies^8^, ^9^, ^11^, ^12^, ^15^, ^25^ in matched/adjusted group and eight studies^8^, ^10^, ^21^, ^23^, ^25^, ^30^, ^31^, ^32^ in unmatched/unadjusted group. Both groups exhibited a significant higher major bleeding rates in MVRm compared with MVRb (HR: 1.20; 95% CI: 1.13−1.28; *p* < .00001; *I*² = 0%; HR: 1.55; 95% CI: 1.30−1.86; *p* < .00001; *I*² = 27%; Figure 4A), which provided a strong evidence of high risk major bleeding in MVRm over MVRb. As for matched/adjusted group of patients under 70 years old,^8^, ^9^, ^11^, ^12^, ^25^ a similar result pooled from five studies could be observed (HR: 1.23; 95% CI: 1.12−1.36; *p* < .0001; *I*²= 0%; Figure 4B).

Figure 4Meta analysis for major bleeding. (A) Forest plot for major bleeding between MVRm and MVRb; (B) Forest plot for major bleeding between MVRm and MVRb for patients aged less than 70 years in matched/adjusted studies; (C) Funnel plot for major bleeding between MVRm and MVRb. CI, confidence interval; MVRb, bioprosthetic mitral valve replacement; MVRm, mechanical mitral valve replacement.

**Figure:**
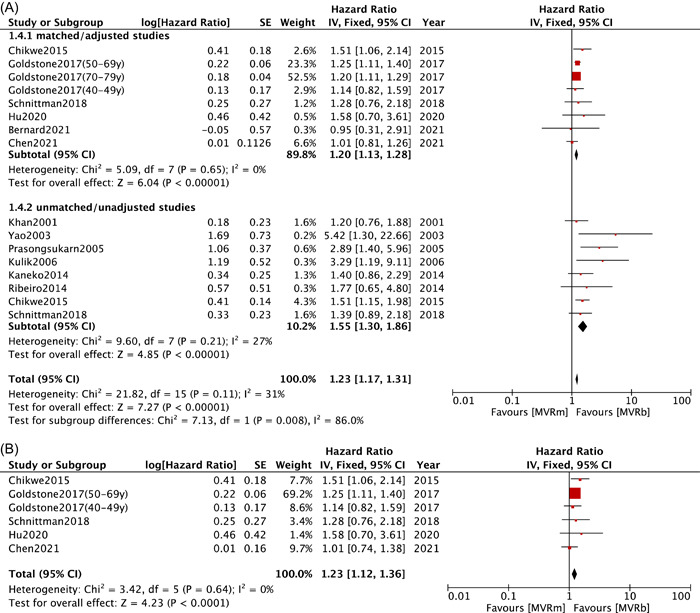

**Figure:**
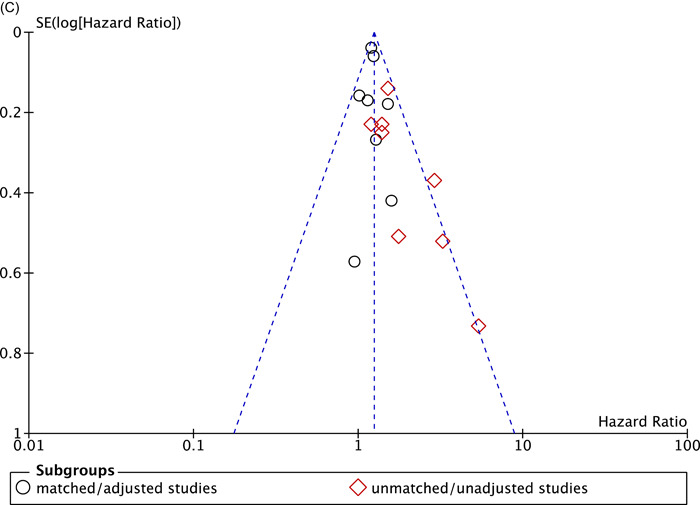

### 2.7 Mitral reoperation

Regarding mitral reoperation, overall estimates collected from six studies^8^, ^9^, ^11^, ^12^, ^25^, ^29^ in matched/adjusted group (HR: 0.34; 95% CI: 0.23−0.50; *p* < .00001; *I*² = 74%; Figure 5A) and eight studies^8^, ^10^, ^21^, ^23^, ^24^, ^25^, ^30^, ^32^ in unmatched/unadjusted group (HR: 0.31; 95% CI: 0.18−0.53; *p* < .0001; *I*² = 77%; Figure 5A) both indicated a significantly greater risk of mitral reoperation following MVRb than that after MVRm. The heterogeneities among studies in two groups were high and a random effects model was applied. For patients aged less than 70 years old, five studies^8^, ^9^, ^11^, ^12^, ^25^ was included for a subgroup analysis showed a significant lower mitral reoperation rates in MVRm compared with MVRb (HR: 0.34; 95% CI: 0.22−0.51; *p* < .00001; *I*² = 61%; Figure 5B). Our meta regression (Table 3) revealed that history of hypertension (*β* = .0346, *p* = .014), DM (*β* = .0911, *p* = .0473) and concomitant CABG procedure (*β*= .0240, *p* = .0006) significantly affected the difference between MVRm and MVRb. However, mean age seemed to affect mitral reoperation despite marginally statistical significance (*β* = .0327, *p* = .0523) (Supporting Information: Figure 2B).

Figure 5Meta analysis for mitral reoperation. (A) Forest plot for mitral reoperation between MVRm and MVRb; (B) Forest plot for mitral reoperation between MVRm and MVRb for patients aged less than 70 years in matched/adjusted studies; (C) Funnel plot for mitral reoperation between MVRm and MVRb. CI, confidence interval; MVRb, bioprosthetic mitral valve replacement; MVRm, mechanical mitral valve replacement.

**Figure:**
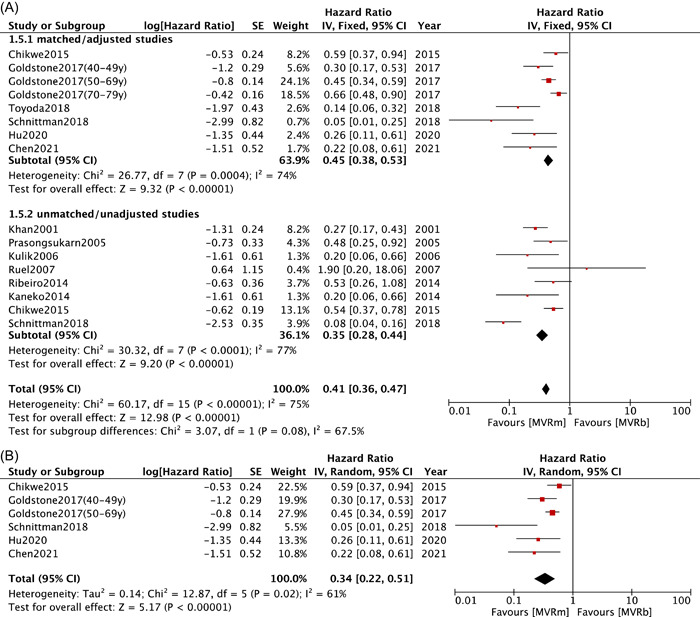

**Figure:**
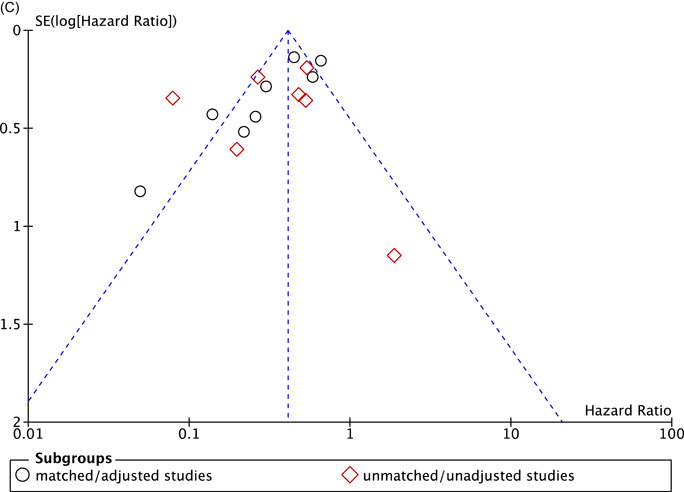

## 3 DISCUSSION

In this meta‐analysis of mitral valve prostheses for MVR patients, long‐term mortality and mitral reoperation were lower among patients who received a mechanical valve than among those who received a bioprosthesis in the mitral position. In contrast, MVRm was associated with a significantly greater risk of major bleeding, stroke or systemic embolism compared with MVRb. The results were consistent with patients up to 70 years of age.

Our study demonstrated no significant difference in operative mortality between MVRm and MVRb in matched/adjusted data among patients up to 70 years old. MVRm showed a significantly lower risk of operative mortality in unmatched/unadjusted group with no heterogeneity among studies (*I*²= 0%), and the funnel plot seemed not asymmetrical (Figure 1C). Considering the unbalanced baseline characteristics of unmatched/unadjusted studies, especially on mean age and comorbidities, which suggested potential publication bias, we cannot arbitrarily advocate MVRm superior to MVRb based on this result. The operative mortality rates could be related to many factors. Confounding bias and variations among studies may have been underestimated and undetected.

This meta‐analysis identified that MVRm provided significantly better long‐term survival outcomes than MVRb among patients both in matched/adjusted studies groups and unmatched/unadjusted groups. We cannot ignore the statistical heterogeneity, which suggested undetected bias underlying in matched/adjusted group. Whereas the baseline characteristics among studies showed no significant variations. Scatters in the funnel plot were almost symmetrical (Figure 2C). Our meta‐regression revealed that older mean age, an increased incidence of CAD, hypertension, and DM seemed to increase the long‐term mortality in MVRb, with significant difference (*p* < .05). The results confirmed that the most critical determinants of survival after valve replacement are age and baseline comorbidity condition.^33^ However, the high‐risk cohort of patients with older age and more complex comorbidities are prone to have a bioprosthesis, considering the shortened life expectancy, high risk for bleeding, and anticoagulation‐related complications.^34^

The long‐term survival benefit that is performed by MVRm comes at the cost of a higher risk of major bleeding, stroke or systemic embolism events in all patients and the subgroup of patients under 70 years of age. Chikwe et al.^8^ reported stroke carried the operative mortality of 8.5%, and major bleeding events were associated with an operative mortality of 7.4%. Goldstone et al.^9^ displayed that both bleeding and stroke events appeared to be associated with higher long‐term mortality. The mortality related to anticoagulation complications may contribute to the advantage in long‐term survival in older patients with MVRb.

The choice between mechanical and biologic MVR is often determined by balancing the risks of anticoagulation and reoperation.^9^ Our data revealed MVRm was associated with a significantly lower mitral reoperation but more significant risk of anticoagulation complications related to major bleeding, stroke or systemic embolism than MVRb. Reoperation was more common among patients who received a biologic prosthesis partially related to structural valve deterioration or failure.^35^ Our meta‐regression demonstrated that a greater proportion of patients with a history of hypertension, DM, and concomitant CABG procedure increased the mitral reoperation rates in MVRb (*p* < .05). As for older patients, mitral reoperation rates seemed to grow, too, despite marginal significance (*p* = .0523). Although the high heterogeneity with asymmetrical funnel plot (Figure 5C) suggested potential confounding bias underlying the result, all included studies of this outcome exhibited a significantly lower mitral reoperation in MVRm than MVRb. The relevant effect size between mitral reoperation and mortality required clarification. Kwedar et al.^36^ reported mitral reoperation carried significant operative mortality of 12.6%. Goldstone et al.^9^ suggested the operative mortality after reoperation of MVR was 14.0%. However, Chikwe et al.^8^ reported operative mortality after reoperation was 5.3%, several studies^37^, ^38^ reported even lower operative mortality and more remarkable functional outcomes followed mitral reoperation. The adverse effect of mitral valve reoperation in contemporary practice seems unclear and needs more investigation for long‐term survival.

Some experienced centers^8^, ^14^ recommended bioprostheses for younger patients of safe outcomes. Our study challenged this assertion and suggested that mechanical mitral valve remains a suitable option in select young patients. The current trend toward abandoning mechanical prostheses in younger patients should be alleviated. During the past decade, there has been a steady increment in biological mitral valves. It now exceeds the use of mechanical prostheses in part to the reports of improved durability of biologic prostheses.^39^ The use of bioprosthesis increased from 16.8% in 1996 to 53.7% in 2013.^9^ As the development of transcatheter mitral valve‐in‐valve replacement or repair, which is a less invasive alternative approach to conventional open surgery for high‐risk patients, mortality associated with reoperation and the use of biologic prostheses proportion in young patients will change.^40^ But the clinical outcome of transcatheter technology remains controversial and needs further evaluation.^41^ Multidisciplinary consideration and careful patient selection are mandatory for this emerging technology.

It is essential for doctors to considering age, comorbidities, life expectancy, quality of life, anticoagulation complications, and mitral reoperation when recommending valve types to patients. Current guidelines have increasingly emphasized patient preference for valve prostheses.^34^ Multiple factors should be combined for an overall consideration in preoperative decision‐making. Our study suggested MVRm was associated with better long‐term survival and lower mitral reoperation rate at the price of higher risks of major bleeding, stroke, or systemic embolism, which should be taken into account for any discussion of mitral valve prosthesis selection.

## 4 LIMITATIONS

There are several limitations of this meta‐analysis that should be mentioned. First, only retrospective studies were involved. One prospective study included only female patients. Such studies are at very high risk for selection bias. Second, the number of studies of patients aged less than 70 years was limited, increasing the risk of selection bias. Third, after matching there is still a difference in terms of associated CABG procedures that are significantly associated with MVRb although with a RR of 0.96. On big numbers as provided by the inclusion of >35 000 patients, this is not a nuance and should be acknowledged as a limitation potentially influencing long‐term survival. Furthermore, we pooled data from studies including patients operated for rheumatic disease, endocarditis, and degenerative disease. Different etiologies of valvular heart disease might affect prosthetic valve selection, which could lead to clinical heterogeneity and is potentially bias. Besides being retrospective data, the studies included in the meta‐analysis have variation generations of the prosthetic valve and significant changes in the medical therapy as in the same period which will have a profound effect on the outcomes. Finally, we extracted clinical outcomes from these studies given the different follow‐up times in this meta‐analysis.

## 5 CONCLUSIONS

MVRm remains a reasonable choice in younger patients for better long‐term survival outcomes and lower mitral reoperation but at the cost of higher risks for major bleeding, stroke or systemic embolism. MVRb may be a better choice over MVRm for patients with a lower life expectancy. With the emerging of transcatheter intervention technologies, mitral valve prosthesis selection may be affected profoundly.

## CONFLICT OF INTEREST

Authors declare no conflict of interest.

## Supporting information

Supporting information.

Supporting information.

## Data Availability

The data that support the findings of this study are available from the corresponding author upon reasonable request.
