# Supplementary material for: Mechanical or biologic prostheses for mitral valve replacement: A systematic review and meta‐analysis
Source: Clin Cardiol. 2022 Jun 5;45(7):701–16. doi: 10.1002/clc.23854 (PMC9286334; doi:10.1002/clc.23854)
Supplement: Supplementary file 1 — Supporting information. [file CLC-45-701-s002.docx]

Embase(n=489), PubMed(n=258), Cochrane library(n=11), Web of science (n=259)

Records identified through database searching

(n=1017)

Records after duplicates removed

(n =439)

Duplicates

(n =578)

Full-text articles assessed for eligibility

(n = 34)

Studies included for Meta-analysis

(n = 22)

Full-text articles excluded, with reasons

(n = 12)

• Case report (n = 1)

• Conference abstract (n = 1)

• No full-text (n = 1)

• Review article (n = 4)

• No comparative outcome (n = 3)

• Combined AVR (n=2)

Irrelevant studies were excluded through title or abstract screening

(n =405)

Supplementary Figure 1. The flowchart of searching process (PRISMA diagram).
