# Supplementary material for: Mechanical or biologic prostheses for mitral valve replacement: A systematic review and meta‐analysis
Source: Clin Cardiol. 2022 Jun 5;45(7):701–16. doi: 10.1002/clc.23854 (PMC9286334; doi:10.1002/clc.23854)
Supplement: Supplementary file 2 — Supporting information. [file CLC-45-701-s001.docx]

A


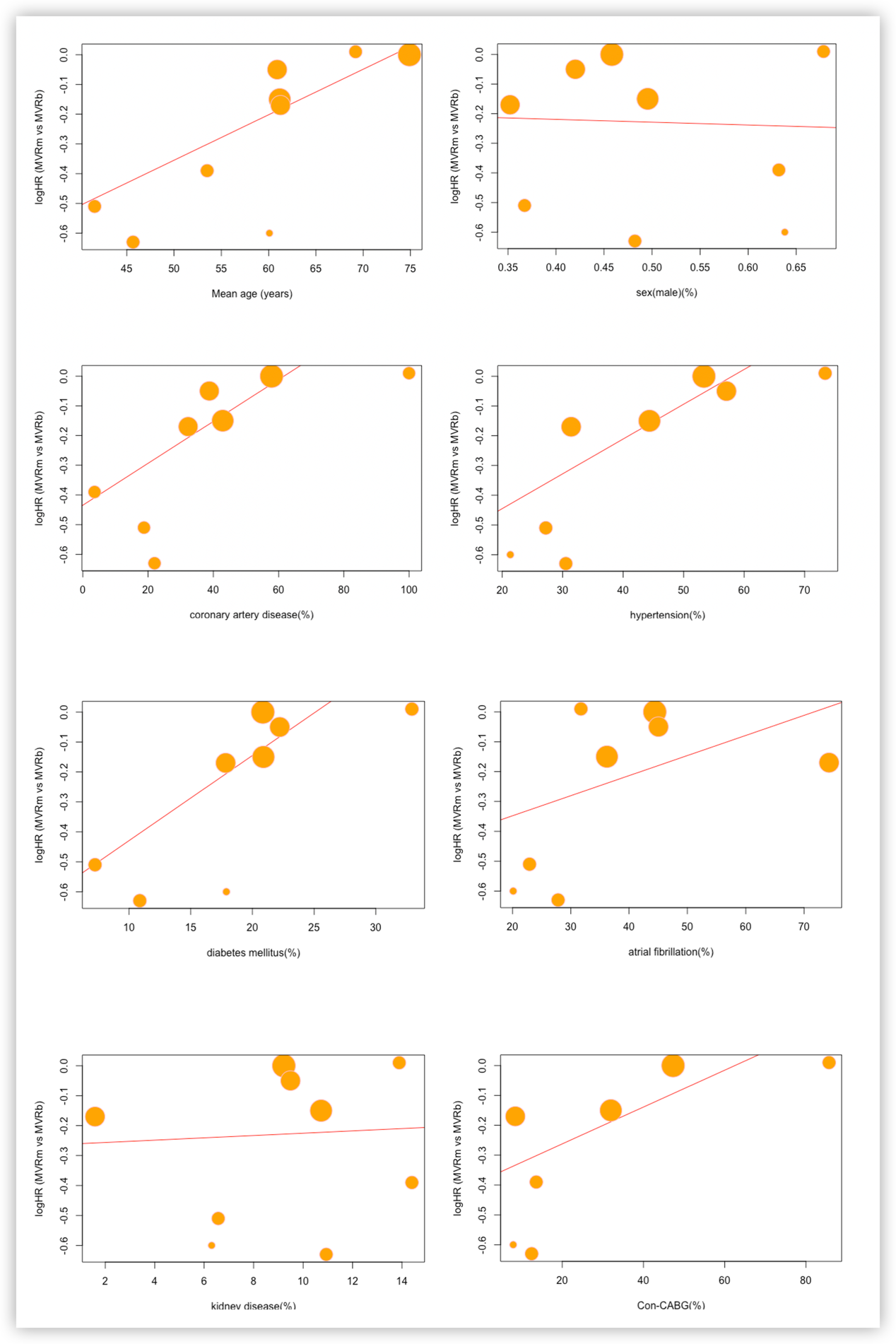


Supplementary figure 2A: Bubble plots of meta-regression in study-covariate against long-term mortality. Con-CABG, concomitant coronary artery bypass grafting. MVRm, mechanical mitral valve replacement; MVRb, bioprosthetic mitral valve replacement. HR, hazard ratio.

B


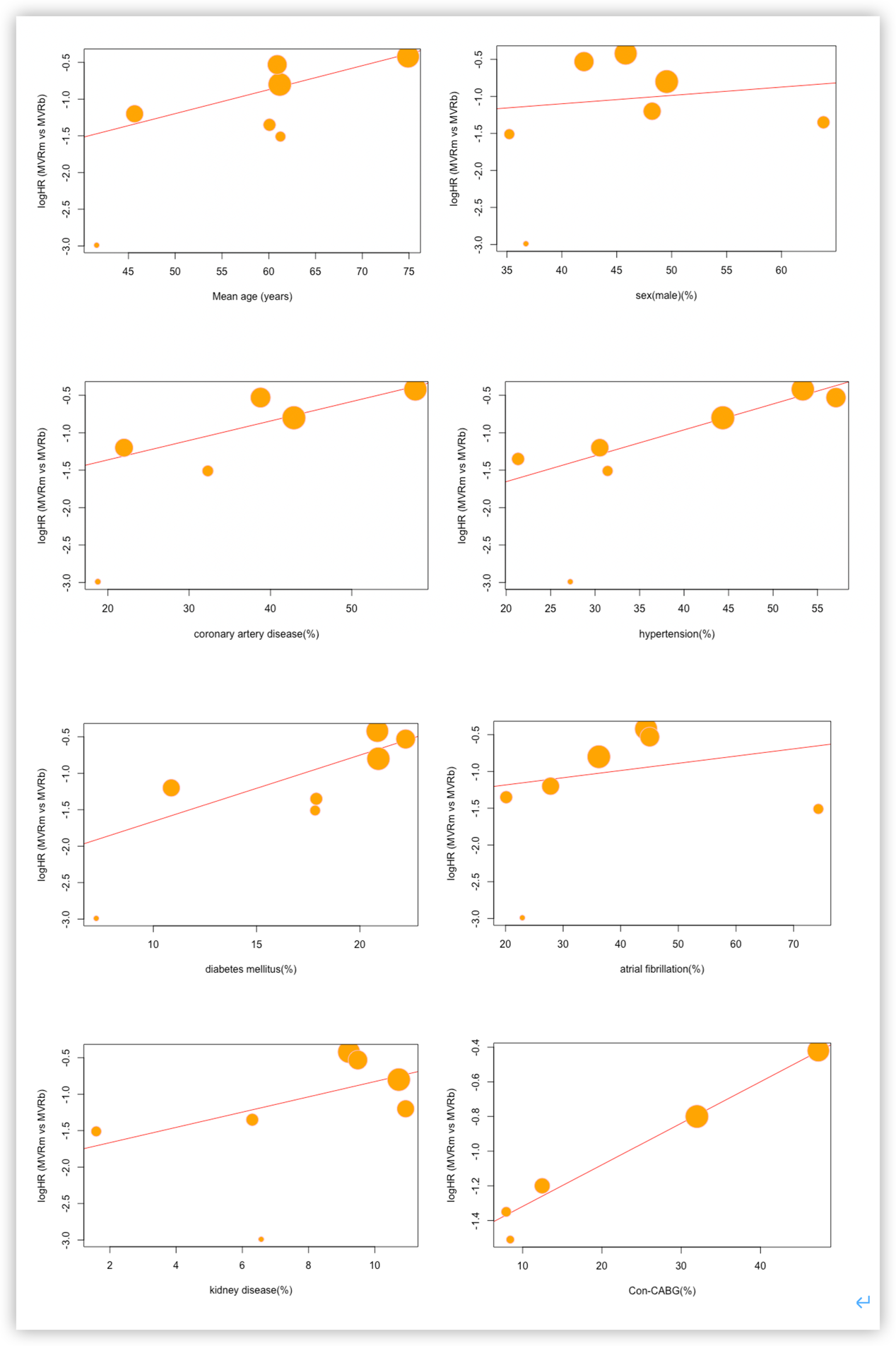


Supplementary figure 2B: Bubble plots of meta-regression in study-covariate against mitral reoperation. Con-CABG, concomitant coronary artery bypass grafting. MVRm, mechanical mitral valve replacement; MVRb, bioprosthetic mitral valve replacement. HR, hazard ratio.
